# Surveillance, Phagocytosis, and Inflammation: How Never-Resting Microglia Influence Adult Hippocampal Neurogenesis

**DOI:** 10.1155/2014/610343

**Published:** 2014-03-19

**Authors:** Amanda Sierra, Sol Beccari, Irune Diaz-Aparicio, Juan M. Encinas, Samuel Comeau, Marie-Ève Tremblay

**Affiliations:** ^1^Ikerbasque Foundation, 48011 Bilbao, Spain; ^2^Achucarro Basque Center for Neuroscience, Bizkaia Science and Technology Park, 48170 Zamudio, Spain; ^3^Department of Neurosciences, University of the Basque Country, 48940 Leioa, Spain; ^4^Centre de Recherche du CHU de Québec, Axe Neurosciences, Canada G1P 4C7; ^5^Département de Médecine Moléculaire, Université Laval, Canada G1V 4G2

## Abstract

Microglia cells are the major orchestrator of the brain inflammatory response. As such, they are traditionally studied in various contexts of trauma, injury, and disease, where they are well-known for regulating a wide range of physiological processes by their release of proinflammatory cytokines, reactive oxygen species, and trophic factors, among other crucial mediators. In the last few years, however, this classical view of microglia was challenged by a series of discoveries showing their active and positive contribution to normal brain functions. In light of these discoveries, surveillant microglia are now emerging as an important effector of cellular plasticity in the healthy brain, alongside astrocytes and other types of inflammatory cells. Here, we will review the roles of microglia in adult hippocampal neurogenesis and their regulation by inflammation during chronic stress, aging, and neurodegenerative diseases, with a particular emphasis on their underlying molecular mechanisms and their functional consequences for learning and memory.

## 1. Microglia: The Resident Immune Cells of the Brain

Microglia were first described in 1919 by the Spanish neuroanatomist Pío del Río Hortega, a disciple of the renowned Santiago Ramón y Cajal, almost half a century later than neurons and astrocytes and just before oligodendrocytes [1]. This delayed appearance into the neuroscience arena is still apparent today, as microglia remain one of the least understood cell types of the brain. Traditionally, microglia were simply considered as “brain macrophages” controlling the inflammatory response during acute insults and neurodegenerative conditions, and only recently was their unique origin revealed. Indeed, microglia were shown to derive from primitive myeloid progenitors of the yolk sac that invade the central nervous system (CNS) during early embryonic development (reviewed in [2]). In contrast, circulating monocytes and lymphocytes, as well as most tissue macrophages, derive from hematopoietic stem cells located initially in the foetal liver and later in the bone marrow [3]. In the adult brain, the microglial population is maintained exclusively by self-renewal during normal physiological conditions [2]. As a consequence, microglia are the only immune cells which permanently reside in the CNS parenchyma, alongside neural tube-derived neurons, astrocytes, and oligodendrocytes.

These past few years, unprecedented insights were also provided into their extreme dynamism and functional behaviour, in health as much as in disease. Indeed, microglia were revealed to be exceptional sensors of their environment, responding on a time scale of minutes to even subtle variations of their milieu, by undergoing concerted changes in morphology and gene expression [4, 5]. During pathological insults, “activated” microglia were particularly shown to thicken and retract their processes, extend filopodia, proliferate and migrate, release factors and compounds influencing neuronal survival (such as proinflammatory cytokines, trophic factors, reactive oxygen species (ROS), etc.), and phagocytose pathogens, degenerating cells and debris, thus providing better understanding of their roles in orchestrating the inflammatory response [6]. These abilities as immune cells are also recruited during normal physiological conditions, where “surveillant” microglia further participate in the remodeling of neuronal circuits by their phagocytic elimination of synapses and their regulation of glutamatergic receptors maturation and synaptic transmission, among other previously unexpected roles [7–9], in addition to their crucial involvement in the phagocytic elimination of newborn cells in the context of adult neurogenesis [10].

**Figure 1:**
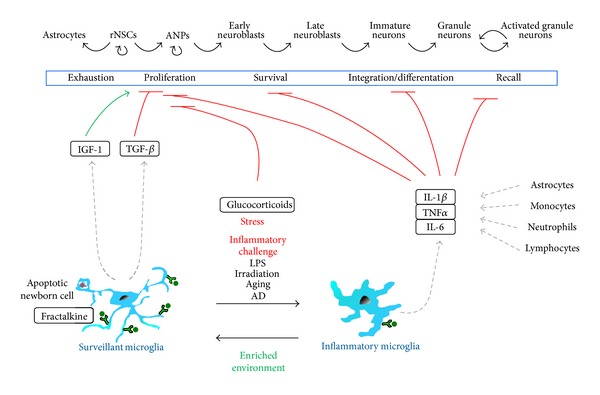
The effects of surveillant and inflammatory microglia on the adult hippocampal neurogenic cascade. During physiological conditions, surveillant microglia effectively phagocytose the excess of apoptotic newborn cells and may release antineurogenic factors such as TGF*β*. This anti-inflammatory state is maintained by neuronal (tethered or released) fractalkine. Enriched environment drives microglia towards a phenotype supportive of neurogenesis, via the production of IGF-1. In contrast, inflammatory challenge triggered by LPS, irradiation, aging, or AD induces the production of proinflammatory cytokines such as IL-1*β*, TNF*α*, and IL-6 by microglia as well as resident astrocytes and infiltrating monocytes, neutrophils, and lymphocytes. These cytokines have profound detrimental effects on adult neurogenesis by reducing the proliferation, survival, integration, and differentiation of the newborn neurons and decreasing their recall during learning and memory paradigms.

Our review will discuss the emerging roles of microglia in adult hippocampal neurogenesis and their regulation by inflammation during chronic stress, aging, and neurodegenerative diseases, with a particular emphasis on their underlying molecular mechanisms and their functional consequences for learning and memory (Figure 1).

## 2. A Brief Overview of Adult Hippocampal Neurogenesis

Adult hippocampal neurogenesis is continuously maintained by the proliferation of neural stem cells located in the subgranular zone (SGZ) [11–13]. These neuroprogenitors have been named “radial glia-like cells” (rNSCs), or type 1 cells, since they morphologically and functionally resemble the embryonic radial glia. They have also been defined as “quiescent neuroprogenitors” because only a small percentage of the population is actively dividing during normal physiological conditions. The lineage of these cells is frequently traced by using analogs of the nucleotide thymidine, such as bromodeoxyuridine (BrdU) which gets incorporated into the DNA of dividing cells during the S phase and can be detected by immunofluorescence. Alternatively, their lineage can be traced by labeling with fluorescent reporters which are delivered to dividing cells by retroviral vectors or expressed by specific cell type promoters via inducible transgenic mice (for a review of the methods commonly used to study adult neurogenesis, see [14]). The daughter cells of rNSCs, also called type 2 cells or amplifying neuroprogenitors (ANPs), rapidly expand their pool by proliferating before becoming postmitotic neuroblasts. Within a month, these neuroblasts differentiate and integrate as mature neurons into the hippocampal circuitry [15]. They however display unique electrophysiological characteristics during several months, being more excitable than mature neurons [16], and constitute a special cell population that is particularly inclined to undergo synaptic remodeling and activity-dependent plasticity [17].

These unique properties of the newborn neurons and the neurogenic cascade in general suggested that adult hippocampal neurogenesis could play an important role in hippocampal-dependent functions that require extensive neuroplasticity such as learning and memory. Indeed, activity-dependent plasticity and learning are long known for modulating adult neurogenesis in a complex, yet specific manner, with adult hippocampal neurogenesis being influenced by learning tasks which depend on the hippocampus [44, 45]. For instance, hippocampal-dependent learning paradigms were found to regulate the survival of newborn neurons, in a positive manner that depends on the timing between their birth and the phases of learning [46, 47]. Young (1.5–2 months old) newborn neurons were also shown to be preferentially activated during memory recall in a water maze task, compared to mature neurons, as determined by colabeling of BrdU with immediate early genes such as c-Fos and Arc, in which expression correlates with neuronal firing [48]. Nonetheless, it has only been in the last few years that loss-of-function and gain-of-function approaches with inducible transgenic mice were able to confirm that adult hippocampal neurogenesis is necessary for synaptic transmission and plasticity, including the induction of long-term potentiation (LTP) and long-term depression [49], as well as trace learning in conditioned protocols [50], memory retention in spatial learning tasks [51, 52], and encoding of overlapping input patterns, that is, pattern separation [53].

Adult hippocampal neurogenesis and its functional implications for learning and memory are however influenced negatively by a variety of conditions that are commonly associated with microglial activation and inflammation in the brain, such as chronic stress, aging, and neurodegenerative diseases, as we will review herein. Indeed, inflammation caused by irradiation produces a sustained inhibition of neurogenesis, notably by decreasing the proliferation and neuronal differentiation of the progenitors, and therefore, exposure to therapeutic doses of cranial irradiation has been widely used for modulating neurogenesis experimentally before the development of more specific approaches [54].

## 3. Regulation of Adult Hippocampal Neurogenesis by Inflammation

Inflammation is a natural bodily response to damage or infection that is generally mediated by proinflammatory cytokines such as interleukin 1 beta (IL-1*β*), interleukin 6 (IL-6), and tumour necrosis factor alpha (TNF*α*), in addition to lipidic mediators such as prostaglandins and leukotrienes. Oftentimes, it is associated with an increased production of ROS, as well as nitric oxide (NO). Together, these proinflammatory mediators lead to an increase in local blood flow, adhesion, and extravasation of circulating monocytes, neutrophils, and lymphocytes [55]. In the brain, microglia are the main orchestrator of the neuroinflammatory response, but other resident cell types, including astrocytes, endothelial cells, mast cells, perivascular and meningeal macrophages, and even neurons, can produce proinflammatory mediators, though perhaps not to the same extent as microglia [56]. In addition, peripheral immune cells invading the CNS during inflammation can further produce proinflammatory mediators, but the respective contribution of microglia versus other cell types in the inflammatory response of the brain is poorly understood.

The harmful effects of inflammation are also widely determined by the actual levels of proinflammatory mediators released, rather than the occurrence or absence of an inflammatory response in itself. For instance, TNF*α* regulates synaptic plasticity by potentiating the cell surface expression of AMPA glutamatergic receptors, thus resulting in a homeostatic scaling following prolonged blockage of neuronal activity during visual system development [57]. However, TNF*α* also produces differential effects at higher concentrations, ranging from an inhibition of long-term potentiation to an enhancement of glutamate-mediated excitotoxicity* in vitro* [58]. Inflammation induced by chronic ventricular infusion of bacterial lipopolysaccharides (LPS; a main component of the outer membrane of Gram-negative bacteria), that is, the most widely used method for inducing an inflammatory challenge, also increases* ex vivo* the hippocampal levels of TNF*α* and IL-1*β*, thereby impairing novel place recognition, spatial learning, and memory formation, but all these cognitive deficits can be restored by pharmacological treatment with a TNF*α* protein synthesis inhibitor, a novel analog of thalidomide, 3,6′-dithiothalidomide [59].

The impact of inflammation on adult hippocampal neurogenesis was originally discovered by Olle Lindvall and Theo Palmer's groups in 2003, showing that systemic or intrahippocampal administration of LPS reduces the formation of newborn neurons in the adult hippocampus, an effect that is prevented by indomethacin, a nonsteroidal anti-inflammatory drug (NSAID) which inhibits the synthesis of proinflammatory prostaglandins [60, 61]. Similarly, inflammation can determine the increase in neurogenesis that is driven by seizures, a context in which neurogenesis can be prevented by LPS and increased by the anti-inflammatory antibiotic minocycline [60]. In these studies, hippocampal proliferation remained unaffected by LPS or minocycline and thus it is likely that inflammation targeted the survival of newborn cells [60, 61], as LPS is known to increase SGZ apoptosis [62]. Inflammation also has further downstream effects on the neurogenic cascade. For instance, LPS increases the number of thin dendritic spines and the expression of the excitatory synapses marker “postsynaptic density protein of 95kDa” (PSD95) in newborn neurons. LPS in addition increases the expression of GABA_A_ receptors at early stages of synapse formation, leading to suggesting a possible imbalance of excitatory and inhibitory neurotransmission in these young neurons [63]. Finally, LPS also prevents the integration of newborn neurons into behaviourally relevant networks, including most notably their activation during spatial exploration, as determined by the percentage of BrdU cells colabeled with the immediate early gene Arc [64].

Importantly, none of these manipulations is specific to microglia and may directly or indirectly affect other brain cells involved in the inflammatory response of the brain. For instance, both LPS and minocycline affect astrocytic function* in vitro* and* in vivo* [65–69]. Furthermore, LPS is known to drive infiltration of monocytes and neutrophils into the brain parenchyma [70]. Monocytes and neutrophils produce major proinflammatory mediators and could therefore act on the neurogenic cascade as well. The implication of microglia in LPS-induced decrease in neurogenesis is nonetheless supported* in vivo* by the negative correlation between the number of newborn neurons (BrdU+, NeuN+ cells) and the number of “activated” microglia (i.e., expressing ED1) [60]. ED1, also called CD68 or macrosialin, is a lysosomal protein which is overexpressed during inflammatory challenge. While the location of ED1 previously suggested its involvement in phagocytosis, its loss of function did not result in phagocytosis deficits and thus, its function still remains unknown (reviewed in [10]). The number of ED1-positive microglia also negatively correlates with neurogenesis during inflammation provoked by cranial irradiation [61]. While correlation does not involve causation, nor can pinpoint to the underlying mechanism, these experiments were the first to reveal a potential role for “activated” microglia in the regulation of adult hippocampal neurogenesis. More direct evidence of microglial mediation in LPS deleterious effects was obtained from* in vitro* experiments, as it was shown that conditioned media from LPS-challenged microglia contained IL-6, which in turn caused apoptosis of neuroblasts [61]. Nonetheless, astrocytes can also release IL-6 when stimulated with TNF*α* or IL-1*β* [71] and chronic astrocytic release of IL-6 in transgenic mice reduced proliferation, survival, and differentiation of newborn cells, thus resulting in a net decrease in neurogenesis [72]. In summary, while the detrimental impact of inflammation on neurogenesis is well established, more work is needed to define the specific roles played by the various inflammatory cells populating the brain.

## 4. Inflammation Associated with Chronic Stress

Across health and disease, the most prevalent condition that is associated with neuroinflammation is “chronic stress,” which commonly refers to the repeated or sustained inability to cope with stressful environmental, social, and psychological constraints. Chronic stress is characterized by an imbalanced secretion of glucocorticoids by the hypothalamic-pituitary-adrenal (HPA) axis (most notably cortisol in humans and corticosterone in rodents), which leads to an altered brain remodeling, massive loss of synapses, and compromised cognitive function [73]. In particular, an impairment of spatial learning, working memory, novelty seeking, and decision making has been associated with chronic stress [74]. Glucocorticoids are well known for their anti-inflammatory properties, as they interfere with NF-*κ*B-mediated cytokine transcription, ultimately delaying wound healing [75]. They are also potent anti-inflammatory mediators* in vivo* [76] and in purified microglia cultures [77]. Recently, repeated administration of high doses of glucocorticoids by intraperitoneal injection, to mimic their release by chronic stress, was also shown to induce a loss of dendritic spines in the motor cortex, while impairing learning of a motor task. A transcription-dependent pathway acting downstream of the glucocorticoid receptor GR was proposed [78, 79] but the particular cell types involved were not identified.

Microglia are considered to be a direct target of the glucocorticoids, as they were shown to express GR during normal physiological conditions* in vivo* [77]. In fact, transgenic mice lacking GR in microglia and macrophages show an increased production of proinflammatory mediators (including TNF*α* and IL-1*β*) and greater neuronal damage in response to an intraparenchymal injection of LPS, compared to wild-type mice [80]. In contrast, glucocorticoids are considered to be proinflammatory in the chronically stressed brain [81], where among other changes they can promote inflammation, oxidative stress, neurodegeneration, and microglial activation [82]. For example, repeated restraint stress induces microglial proliferation and morphological changes, including a hyperramification of their processes in the adult hippocampus following restraint stress [83], but a nearly complete loss of processes in the context of social defeat [84]. Prenatal restraint stress also causes an increase in the basal levels of TNF*α* and IL-1*β*, while increasing the proportion of microglia showing a reactive morphology in the adult hippocampus [85]. Similarly, social defeat leads to an enhanced response to the inflammatory challenge induced by intraperitoneal injection of LPS, including an increased production of TNF*α* and IL-1*β*, and expression of inducible NO synthase (iNOS) by microglia, accompanied by an increased infiltration of circulating monocytes [84, 86]. Therefore, microglia are a strong candidate for mediating some of the effects of stress on adult neurogenesis, as will be discussed below, in synergy with other types of inflammatory cells.

Chronic stress is well known for its negative effects on hippocampal neurogenesis (reviewed in [87, 88]), although not all stress paradigms are equally effective [89]. Several stress paradigms can decrease neuroprogenitors proliferation in the tree shrew [90] and in mice [91, 92], although this effect seems to be compensated by an increased survival of newborn neurons [92] and whether stress results in a net increase or decrease in neurogenesis remains controversial (reviewed in [87, 88]). The effects of stress on adult neurogenesis seem to be mediated at least partially by glucocorticoids, because mice lacking a single copy of the GR gene show behavioural symptoms of depression including learned helplessness, neuroendocrine alterations of the HPA axis, and impaired neurogenesis [93]. In parallel, chronic stress is associated with an increased inflammatory response, which may inhibit neurogenesis as well. For instance, serum levels of IL-1*β* and IL-6 are significantly increased in depressed patients [94]. In mice, restraint stress leads to a widespread activation of NF-*κ*B in the hippocampus, including at the level of neuroprogenitors [95] and increased protein levels of IL-1*β* [96]. In addition to the direct role of glucocorticoids, IL-1*β* also seems to mediate some of the effects of mild chronic stress, because* in vivo* manipulations that block IL-1*β* (either pharmacologically or in null transgenic mice) prevent the anhedonic stress response and the antineurogenic effect of stress [91, 96]. Moreover, the corticoids and IL-1*β* pathways may regulate each other in a bidirectional manner because the administration of a GR antagonist can blunt the LPS-induced production of hippocampal IL-1*β* in stressed mice [97], whereas mice knockout for the IL-1*β* receptor (IL-1R1) fail to display the characteristic elevation of corticosterone induced by mild chronic stress [96]. Another stress-related cytokine, IL-6, induces depressive phenotypes and prevents the antidepressant actions of fluoxetine when administered to mice* in vivo* [98]. So far the effects of stress on neurogenesis via corticosteroids and inflammation have been assumed to be cell autonomous, as neuroprogenitors express both GR [99] and IL-1R1 [95]. The potential participation of microglia is yet to be determined, but there are some reports of a direct effect of stress on microglial activation. For instance, microglia acutely isolated from mice subjected to acute stress (by inescapable tail shock) showed a primed response to LPS challenge by producing higher levels of IL-1*β* mRNA* ex vivo* [100], and the specific loss of expression of GR in microglia leads to a blunted inflammatory response* in vitro* and to a decreased neuronal damage* in vivo* in response to LPS [80]. In stress paradigms, these enhanced responses of microglia to inflammatory challenges are similar to their age-related “priming” which has been associated with and is possibly due to an increased basal production of proinflammatory mediators. However, whether microglia express increased levels of IL-1*β* and other proinflammatory cytokines in response to stressful events is presently unclear [101]. It is thus possible that some of the antineurogenic effects of stress are exerted by means of microglial-dependent inflammation, but this hypothesis remains to be experimentally tested.

## 5. Inflammation Associated with Aging and Neurodegenerative Diseases 

Inflammation is also commonly associated with normal aging and neurodegenerative diseases and, therefore, could represent a putative underlying mechanism that explains their decrease in hippocampal neurogenesis. Nonetheless, inflammation is also associated with neurological diseases, such as epilepsy or stroke, where neurogenesis is thought to be increased, although the data from rodents and humans is somewhat conflictive [102]. Neurogenesis is well known to decline throughout adulthood and normal aging in rodents and humans [103, 104], but the decay is more pronounced and occurs later in life in mice than in humans [105]. The aging-associated decrease in neurogenesis has been shown to occur mainly as a consequence of exhaustion of the rNSC population which, after being recruited and activated, undergo three rounds of mitosis in average and then terminally differentiate into astrocytes [12, 106]. In addition, a reduced mitotic capacity of the neuroprogenitors could further contribute to decreasing neurogenesis [106], and moreover, an age-related increase in the levels of proinflammatory cytokines could also hinder neurogenesis in the aging brain. Serum levels of IL-1*β*, IL-6, and TNF*α* are elevated in elderly patients [107, 108]. Aged microglia express higher levels of these proinflammatory cytokines and show a greater response to LPS inflammatory challenge, that is, a “primed” response, than their younger counterparts [109]. The origin of this low-grade age-related inflammation (“inflamm-aging” [110]) remains unknown and may be related to both aging and damage to the surrounding neurons, as well as aging of the immune system* per se*.

At the cellular level, stress to the endoplasmic reticulum (ER) caused by various perturbations, such as nutrient depletion, disturbances in calcium or redox status, or increased levels of misfolded proteins, can induce a cell-autonomous inflammatory response to neurons. Stress to the ER, a multifunctional organelle which is involved in protein folding, lipid biosynthesis, and calcium storage triggers a homeostatic response mechanism named the unfolding protein response (UPR), aiming to clear the unfolded proteins in order to restore normal ER homeostasis [111]. However, if the ER stress cannot be resolved, the UPR also initiates inflammatory and apoptotic pathways via activation of the transcription factor NF-*κ*B which controls the expression of most proinflammatory cytokines [112]. In the brain, ER stress is often initiated by the formation of abnormal protein aggregates in several neurodegenerative diseases such as Alzheimer's disease (AD), Parkinson's disease (PD), amyotrophic lateral sclerosis (ALS), Huntington's disease (HD), and prion-related disorders [113]. This neurodegeneration-associated ER stress is assumed to occur mostly in neurons, but there are some examples of microglial protein misfolding as well. For instance, both microglia and neurons overexpress CHOP (C/EBP homologous protein), a transcription factor which is activated during ER stress in human patients and mouse models of ALS [114]. Inflammation has been speculated to be a main negative contributor to the pathology of ALS [115], but a direct microglial involvement in mediating the inflammatory response to abnormal protein aggregation in ALS and other neurodegenerative conditions remains to be tested. Finally, ER stress has been linked to a variety of inflammatory conditions [116, 117], including chronic stress, diet-induced obesity, and drug abuse, as well as atherosclerosis and arthritis [118–120]. During normal aging, a progressive decline in expression and activity of key ER molecular chaperones and folding enzymes could also compromise the adaptive response of the UPR, thereby contributing to the age-associated decline in cellular functions [118]. Therefore, aging is strongly associated with a chronic ER stress which leads to increased activation of NF-*κ*B [112]; however, the contribution of the different brain cell types to “inflamm-aging” is still poorly understood. The detrimental effects on neurogenesis of increased proinflammatory cytokines in the aging brain are not necessarily related to microglia, but also to stressed neurons. Furthermore, ER stress may also cause a cell-autonomous response in neural stem cells [121], although its impact on neurogenesis remains to be experimentally determined.

In addition, aging is accompanied by an increased level of mitochondrial oxidative stress, which in turn activates the “Inflammasome” [122], a group of multimeric proteins comprising the interleukin 1 converting enzyme (ICE, caspase 1) which serves to release the active form of the cytokine [123]. IL-1*β* may act directly on rNSCs (visualised by labeling with the Sox2 marker), as they express IL-1R1 in the adult hippocampus [91]. Treatment with IL-1*β* decreases hippocampal proliferation in young mice [91] and pharmacological inhibition of ICE partially restores the number of newborn neurons in aged mice without significantly affecting their differentiation rate [124]. Transgenic IL-1*β* overexpression results in chronic inflammation and depletion of doublecortin-labeled neuroblasts, thus mimicking the aging-associated depletion of neurogenesis [125]. The actual mechanism of action of IL-1*β* on neurogenesis in aged mice, including decreased proliferation of rNSCs/ANPs and survival of newborn neurons, remains undetermined. Microglia are a main source of IL-1*β* in the aging brain, but the hypothesis that microglia-derived IL-1*β* is responsible for depleting neurogenesis in the aging brain remains to be directly tested.

The regulation of neurogenesis by IL-1*β* in the aging brain has been further linked to the activity of another cytokine, the chemokine fractalkine, or CX3CL1. Fractalkine has soluble and membrane-tethered forms and is exclusively expressed by neurons, while the fractalkine receptor (CX3CR1) is expressed in the brain by microglia alone [126]. This module forms a unique neuron-microglia signalling unit that controls the extent of microglial inflammation in several neurodegenerative conditions including PD, ALS [127], or AD [128]. In fact, CX3CR1 blocking antibodies increase the production of hippocampal IL-1*β* when administered to young adult rats [129]. Importantly, chronic treatment with fractalkine increases hippocampal proliferation and the number of neuroblasts in aged (22 months old) but not young (3 months old) or middle-aged rats (12 months old), whereas an antagonists of CX3CR1 has the opposite effects in young, but not in middle-aged nor old rats [129]. Since fractalkine expression is decreased during aging [129], a reduced neuron-microglia signalling might be releasing the brake on microglial contribution to inflammatory responses, although increased levels of fractalkine were instead reported in aged rat hippocampus by other studies [68]. Additional insights into the role of fractalkine signalling come from knock-in mice in which the endogenous CX3CR1 locus is replaced by the fluorescent reporter GFP [126]. The initial studies suggested that CX3CR1^GFP/GFP^ (i.e., CX3CR1^−/−^) mice have no significant differences in brain development and functions [130], but more systematic investigations recently revealed a long list of hippocampal-dependent changes in young (3 months old) CX3CR1^GFP/GFP^ and CX3CR1^GFP/+^ mice compared to wild-type mice. These changes notably included decreased neuroprogenitors proliferation and neuroblasts number, impaired LTP, performance in contextual fear conditioning and water maze spatial learning and memory, and, importantly, increased IL-1*β* protein levels [131]. The signalling pathway of fractalkine-IL-1*β* is functionally relevant, because IL-1R1 antagonists rescued LTP and cognitive function in CX3CR1^GFP/GFP^ mice [131]. In sum, even though neuronal fractalkine seems to be sufficient for restraining the inflammatory activity of microglia in young rats, its downregulation during aging could activate the microglial inflammatory response and thereby subsequently reduce the proliferation of remaining neuroprogenitors.

In AD, inflammatory cytokines such as IL-1*β* are overexpressed in the microglia associated with the amyloid beta (A*β*) plaques of postmortem samples [132] and in transgenic mice modeling the disease [133]. The loss of synapses (from hippocampus to frontal cortex) is one of the main pathological substrates in this disease, but adult neurogenesis is also severely reduced in most mouse models of AD, possibly due to a decreased proliferation of neuroprogenitors and a decreased survival of newborn cells, even though the putative changes in the neurogenic cascade in postmortem samples remain controversial (reviewed in [102]). This lack of agreement is possibly explained by the fact that the vast majority of AD cases have a late onset over 65 years of age, when little neurogenesis remains. In contrast, in most transgenic AD mouse models, the A*β* accumulation, cognitive deficits, and changes in neurogenesis are already detectable in young animals (2-3 months old). The study of AD is further hindered by the difficulty in comparing the time course and pathology across different mouse models. For instance, early treatment with minocycline can improve cognition and reduce A*β* burden in mice expressing the human amyloid precursor protein (APP) [134]. In contrast, in mice expressing APP and a mutated form of presenilin 1 (PS1), which is part of the *γ* secretase pathway that cleaves A*β*, inflammation is reduced without any detectable changes in A*β* plaques deposition [135]. Concomitantly with a decrease in tissue inflammatory cytokines and number of microglial cells, minocycline restores neurogenesis and hippocampus-dependent memory deficits in these APP/PS1 mice [135], indirectly suggesting that cognitive decay in AD may be at least in part related to a detrimental effect of inflammation on hippocampal neurogenesis. Direct evidence that neurogenesis is associated with the cognitive performance in AD is still lacking. Further research is also necessary to determine the neurogenic targets of AD-related inflammation. One central open question for future therapies aiming at increasing neurogenesis and cognition in AD is whether neuroprogenitors are spared or whether their age-induced loss becomes accelerated. Rather than increasing the proliferation and neurogenic output of the few rNSCs remaining in an old AD brain, it may be more relevant to develop strategies that prevent the age-related loss of neuroprogenitors in presymptomatic patients.

In summary, inflammation associated with a wide variety of experimental models of disease produces strong detrimental effects on hippocampal neurogenesis. These effects on human neurogenesis are however not so well described and,* in vitro*, IL-1*β* increases the proliferation of hippocampal embryonic neuroprogenitors but decreases their differentiation into neurons [136]. Novel methods to assess hippocampal neurogenesis in the living human brain, from metabolomics of neuroprogenitors to hippocampal blood brain volume (reviewed in [102]), will help to determine the contribution of inflammation to adult neurogenesis in the healthy and diseased human brain during aging.

## 6. Normal Physiological Conditions 

In the healthy mature brain, microglia are an essential component of the neurogenic SGZ niche, where they physically intermingle with neuroprogenitors, neuroblasts, and newborn neurons [62]. Here, surveillant microglia effectively and rapidly phagocytose the excess of newborn cells undergoing apoptosis [62]. Importantly, microglial phagocytosis in the adult SGZ is not disturbed by inflammation associated with aging or by LPS challenge, as the phagocytic index (i.e., the proportion of apoptotic cells completely engulfed by microglia) is maintained over 90% in these conditions [62]. Nonetheless, the consequences of microglial phagocytosis on adult hippocampal neurogenesis remain elusive. Treatment of mice with annexin V, which binds to the phosphatidylserine (PS) receptor and prevents the recognition of PS on the surface of apoptotic cells, presumably blocking phagocytosis, increases the number of apoptotic cells in the SGZ [40]. Concomitantly, annexin V reduces neurogenesis by decreasing the survival of neuroblasts without affecting neuroprogenitors proliferation [40]. Similar results were obtained in transgenic mice knock-out for ELMO1, a cytoplasm protein which promotes the internalization of apoptotic cells, although the effects on neurogenesis were ascribed to a decreased phagocytic activity of neuroblasts [40]. The actual phagocytic target of the neuroblasts remains undetermined, but the newborn apoptotic cells in the adult SGZ are exclusively phagocytosed by microglia, at least in physiological conditions [62]. Nevertheless, none of the above manipulations has specifically tested the role of microglial phagocytosis in hippocampal-dependent learning and memory and thus, the functional impact of microglial phagocytosis in adult neurogenic niches during normal physiological conditions remains to be elucidated.

Microglial phagocytosis of apoptotic cells is actively anti-inflammatory, at least* in vitro*, and thus it has been hypothesized that anti-inflammatory cytokines produced by phagocytic microglia may further regulate neurogenesis [10]. For instance, transforming growth factor beta (TGF*β*), which is produced by phagocytic microglia* in vitro* [137], inhibits the proliferation of SGZ neuroprogenitors [138]. Microglia are further able to produce proneurogenic factors* in vitro* [139]. When primed with cytokines associated with T helper cells such as interleukin 4 (IL-4) or low doses of interferon gamma (IFN*γ*), cultured microglia support neurogenesis and oligodendrogenesis through decreased production of TNF*α* and increased production of insulin-like growth factor 1 (IGF-1) [139], an inducer of neuroprogenitor proliferation [26]. A list of potential factors produced by microglia and known to act on neuroprogenitor proliferation can be found in Table 1. In addition, recent observations suggest that neuroprogenitor cells may not only regulate their own environment, but also influence microglial functions. For instance, vascular endothelial growth factor (VEGF) produced by cultured neuroprecursor cells directly affects microglial proliferation, migration, and phagocytosis [20]. More potential factors produced by neuroprogenitors shown to be influencing microglial activity and function can be found in Table 2. However, it has to be taken into account that most of these observations were obtained in culture and that further research is needed in order to elucidate whether those factors are also secreted and have the same regulatory responses* in vivo*.

In addition, microglial capacity to remodel and eliminate synaptic structures during normal physiological conditions has suggested that microglia could also control the synaptic integration of the newborn neurons generated during adult hippocampal neurogenesis [140]. Three main mechanisms were proposed: (1) the phagocytic elimination of nonapoptotic axon terminals and dendritic spines, (2) the proteolytic remodeling of the perisynaptic environment, and (3) the concomitant structural remodeling of dendritic spines [7, 140]. Indeed, microglial contacts with synaptic elements are frequently observed in the cortex during normal physiological conditions, sometimes accompanied by their engulfment and phagocytic elimination [141–143], as in the developing retinogeniculate system [144]. Microglial cells are distinctively surrounded by pockets of extracellular space, contrarily to all the other cellular elements [142], suggesting that microglia could remodel the volume and geometry of the extracellular space, and thus the concentration of various ions, neurotransmitters, and signalling molecules in the synaptic environment. Whether microglia create the pockets of extracellular space themselves or not remains unknown, but these pockets could result from microglial release of extracellular proteases such as metalloproteinases and cathepsins [145], which are well known for influencing the formation, structural remodeling, and elimination of dendritic spines* in situ* and also experience-dependent plasticity* in vivo* [7, 146]. More recently, microglial phagocytosis of synaptic components was also observed in the developing hippocampus, in the unique time window of synaptogenesis, a process which is notably regulated by fractalkine-CX3CR1 signalling [147]. Therefore, the attractive hypothesis that microglial sculpts the circuitry of newborn cells in the adult hippocampus deserves further attention.

Lastly, microglia were also involved in increasing adult hippocampal neurogenesis in the enriched environment (EE) experimental paradigm. EE is a paradigm mimicking some features of the normal living circumstances of wild animals, as it gives them access to social interactions, toys, running wheels, and edible treats. EE has long been known to enhance neurogenesis by acting on newborn cells survival, resulting ultimately in an enlargement of the dentate gyrus [148]. Functionally, these changes are accompanied by enhanced spatial learning and memory formation with the water maze paradigm [149]. Similar increases in neurogenesis are obtained by subjecting mice to voluntary running paradigms, although in this case the effect is mediated by increased neuroprogenitor proliferation [150]. During inflammatory conditions, EE is antiapoptotic and neuroprotective [151] and it limits the hippocampal response to LPS challenge by decreasing the expression of several cytokines and chemokines, including IL1-*β* and TNF*α* [152]. In fact, EE is believed to counteract the inflammatory environment and rescue the decreased number of neuroblasts in CX3CR1^GFP/GFP^ mice compared to wild-type mice [153]. The effects of EE are independent of the IL-1*β* signalling pathway, as it increases neurogenesis in mice that are null for IL-1R1 [154]. EE also induces microglial proliferation and expression of the proneurogenic IGF-1 [155], but the full phenotype of microglia in EE compared to standard housing and its impact on the neurogenic cascade remains to be determined.

The mechanisms behind the anti-inflammatory actions of EE are unknown, but they were suggested to involve microglial interactions with T lymphocytes through an increased expression of the major histocompatibility complex of class II (MHC-II) during EE [155]. MHC-II is responsible for presenting the phagocytosed and degraded antigens to the antibodies expressed on the surface of a subtype of T lymphocytes (T helper or CD4+ cells), thus initiating their activation and production of antigen-specific antibodies. Severe combined immunodeficient (SCID) mice lacking either T and B lymphocytes or nude mice lacking only T cells have impaired proliferation and neurogenesis in normal and EE housing compared to wild-type mice [155], as well as impaired performance in the water maze [156]. Similarly, antibody-based depletion of T helper lymphocytes impairs basal and exercise-induced proliferation and neurogenesis [157]. Furthermore, a genetic study in heterogeneous stock mice, which descend from eight inbred progenitor strains, has found a significant positive correlation between genetic loci associated to hippocampal proliferation and to the proportion of CD4+ cells among blood CD3+ lymphocytes [158]. Additional experiments are needed to fully determine the possible interactions between microglia and T cells in neurogenesis, because, at least in normal physiological conditions, (1) T cell surveillance of the brain parenchyma is minimal, (2) microglia are poor antigen presenting cells, and (3) antigen presentation by means of MHC-II family of molecules is thought to occur outside the brain, that is, in the meninges and choroid plexus [159]. In fact, during voluntary exercise, there are no significant changes in T cell surveillance of the hippocampus, nor a direct interaction between T cells and microglia, nor any changes in the gene expression profile of microglia, including that of IGF-1, IL-1*β*, and TNF*α* [160]. The number of microglia is also inversely correlated with the number of hippocampal proliferating cells, rNSCs, and neuroblasts in aged (8 months) mice subjected to voluntary running, as well as* in vitro* cocultures of microglia and neuroprogenitors, which has been interpreted as resulting from an overall inhibitory effect of microglia on adult neurogenesis [161]. Even though EE is clearly a more complex environmental factor than voluntary running, further research is necessary to disregard nonspecific or indirect effects of genetic or antibody-based T cells depletion on microglia and other brain cell populations, including rNSCs. For instance, adoptive transfer of T helper cells treated with glatiramer acetate, a synthetic analog of myelin basic protein (MBP) approved for the treatment of multiple sclerosis, produces a bystander effect on resident astrocytes and microglia by increasing their expression of anti-inflammatory cytokines such as TGF*β* [162]. Alternatively, it has been suggested that T cells may mediate an indirect effect on adult hippocampal neurogenesis by increasing the production of brain-derived neurotrophic factor (BDNF) [157], which is involved in the proneurogenic actions of EE [163]. Whether BDNF can counteract the detrimental effects of T cell depletion on neurogenesis remains unknown. Overall, the roles of microglia in EE and running-induced neurogenesis are unclear and have to be addressed with more precise experimental designs. In summary, surveillant microglia are part of the physical niche surrounding the neural stem cells and newborn neurons of the mature hippocampus, where they continuously phagocytose the excess of newborn cells. Microglia were also linked to the proneurogenic and anti-inflammatory effects of voluntary running and EE, but direct evidence is missing. The overall contribution of microglia to neurogenesis and learning and memory in normal physiological conditions remains largely unexplored at this early stage in the field.

## 7. Conclusion

In light of these observations, microglia are now emerging as important effector cells during normal brain development and functions, including adult hippocampal neurogenesis. Microglia can exert a positive or negative influence on the proliferation, survival, or differentiation of newborn cells, depending on the inflammatory context. For instance, microglia can compromise the neurogenic cascade during chronic stress, aging, and neurodegenerative diseases, by their release of proinflammatory cytokines such as IL-1*β*, IL-6, and TNF*α*. A reduced fractalkine signalling between neurons and microglia could also be involved during normal aging. However, microglia are not necessarily the only cell type implicated because astrocytes, endothelial cells, mast cells, perivascular and meningeal macrophages, and to a lesser extent neurons and invading peripheral immune cells could further contribute by releasing proinflammatory mediators.

Additionally, microglia were shown to phagocytose the excess of newborn neurons undergoing apoptosis in the hippocampal neurogenic niche during normal physiological conditions, while a similar role in the synaptic integration of newborn cells was also proposed in light of their capacity to phagocytose synaptic elements. Lastly, microglial interactions with T cells, leading to the release of anti-inflammatory cytokines, neurotrophic factors, and other proneurogenic mediators (notably during EE and voluntary running), could counteract the detrimental effects of inflammation on adult hippocampal neurogenesis and their functional implications for learning and memory.

However, further research is necessary to assess the relative contribution of microglia versus other types of resident and infiltrating inflammatory cells and to determine the nature of the effector cytokines and other inflammatory mediators involved, as well as their cellular and molecular targets in the neurogenic cascade. Such research will undoubtedly help to develop novel strategies aiming at protecting the neurogenic potential and ultimately its essential contribution to learning and memory.

## Figures and Tables

**Table 1:** Summary of factors secreted by microglia and the potential effect they have on neuroprogenitors *in vitro*.

Microglia secreted factors	Reference	Modulation of neural progenitor cells	Reference
BDNF	[18]	Differentiation	[19]
EGF	[20]	Survival, expansion, proliferation, differentiation	[21]
FGF*β*	[22]	Survival and expansion	[23]
GDNF	[24]	Survival, migration, and differentiation	[25]
IGF-1	[21]	Proliferation	[26]
IL-1*β*	[27]	Reduction in migration	[27]
IL-6	[28]	Inhibition of neurogenesis	[29]
IL-7	[20]	Differentiation	[30]
IL-11	[20]	Differentiation	[30]
NT-4	[24]	Differentiation	[31]
PDGF	[32]	Expansion and differentiation	[33]
TGF*β*	[34]	Inhibition of proliferation	[19]

**Table 2:** Summary of factors secreted by neuroprogenitors and the potential effect they have on microglia *in vitro*.

NPC secreted factors	Reference	Modulation of microglia	Reference
BDNF	[18]	Proliferation and induction of phagocytic activity	[35]
Haptoglobin	[24]	Neuroprotection	[36]
IL-1*β*	[37]	Intracellular Ca^+2^ elevation and proliferation	[22]
IL-6	[37]	Increase in proliferation	[38]
M-CSF	[20]	Mitogen	[39]
NGF	[40]	Decrease in LPS-induced NO	[41]
TGF*β*	[37]	Inhibition of TNF*α* secretion	[42]
TNF*α*	[37]	Upregulation of IL-10 secretion	[43]
VEGF	[20]	Induction of chemotaxis and proliferation	[20]

## Abbreviations

AD: Alzheimer's disease
ANPs: Amplifying neuroprogenitors
APP: Amyloid precursor protein
A*β*: Amyloid beta
BDNF: Brain-derived neurotrophic factor
BrdU: 5-Bromo-2′-Deoxyuridine
CX3CL1: Fractalkine
CX3CR1: Fractalkine receptor
EAE: Experimental acute encephalomyelitis
EE: Enriched environment
EGF: Epidermal growth factor
FGFb: Basic fibroblast growth factor
GDNF: Glial cell line-derived neurotrophic factor
GFAP: Glial fibrillary acidic protein
GR: Glucocorticoid receptor
HPA: Hypothalamic-pituitary-adrenal axis
ICE: Interleukin 1 converting enzyme
IL-1*β*: Interleukin 1 beta
IL-1R1: Interleukin 1 beta receptor
IL-4: Interleukin 4
IL-6: Interleukin 6
IL-7: Interleukin 7
IL-11: Interleukin 11
IFN*γ*: Interferon gamma
IGF-1: Insulin-like growth factor 1
iNOS: Inducible nitric oxide synthase
LPS: Bacterial lipopolysaccharides
LTP: Long term potentiation
M-CSF: Macrophage colony-stimulating factor
MBP: Myelin basic protein
MHC-II: Major histocompatibility complex class II
MOG: Myelin oligodendrocyte glycoprotein
NF-*κ*B: Nuclear factor kappa-light-chain-enhancer of activated B cells
NGF: Nerve growth factor
NO: Nitric oxide
NSAID: Nonsteroidal anti-inflammatory drug
NT-4: Neurotrophin-4
PDGF: Platelet-derived growth factor
PS: Phosphatidylserine
PS1: Presenilin 1
ROS: Radical oxygen species
SCID: Severe combined immunodeficiency
SGZ: Subgranular zone
TGF*β*: Transforming growth factor beta
TNF*α*: Tumor necrosis factor alpha
VEGF: Vascular endothelial growth factor.
